# Dairy and plant based food intakes are associated with altered faecal microbiota in 2 to 3 year old Australian children

**DOI:** 10.1038/srep32385

**Published:** 2016-10-03

**Authors:** P. Smith-Brown, M. Morrison, L. Krause, P. S. W. Davies

**Affiliations:** 1 Children’s Nutrition Research Centre, Child Health Research Centre, The University of Queensland, Australia; 2 The University of Queensland Diamantina Institute, Translational Research Institute, The University of Queensland, Australia

## Abstract

The first 1000 days (conception to 24 months) is when gut microbiota composition and eating patterns are established, and a critical period influencing lifelong health. The aim of this study is to examine the associations between food intakes and microbiota composition at the end of this period. Diet was quantified for 37 well-nourished Australian children aged between 2 to 3 years by using a food frequency questionnaire and 24 hr recalls. Both dairy and plant-based (fruit, vegetables, soy, pulses and nuts) food intakes were associated with distinct microbiota profiles. Dairy intake was positively associated with the Firmicutes:Bacteroidetes ratio, and in particular *Erysipelatoclostridium* spp., but negatively associated with species richness and diversity. Vegetable intake was positively associated with the relative abundance of the *Lachnospira* genus, while soy, pulse and nut intake was positively associated with the relative abundance of bacteria related to *Bacteroides xylanisolvens*. Fruit intake, especially apples and pears, were negatively associated with the relative abundance of bacteria related to *Ruminococcus gnavus.* In this cohort of young children dairy and plant based food intakes were found to be associated with altered microbiota composition. Further exploration is needed to elucidate the effect of these dietary and microbial differences on host phenotype.

The period from conception to age 24 months has been identified as the critical window of opportunity in which good nutrition and healthy growth have lasting benefits throughout life and into subsequent generations^1^,^2^. This window of opportunity has been named “the first 1000 days” (thousanddays.org) and corresponds to the period of developmental plasticity in which the human system is adaptable and sensitive to the environment, followed by a loss of plasticity and fixed function^3^,^4^. The concept that stimuli or insults within this critical period of development can determine lifelong risk of metabolic disease has been termed Developmental Origins of Health and Disease (DOHaD)^5^ or nutritional programming^6^.

It is proposed that important mechanisms for the translation of early diet to lifelong health are through modification of the epigenome and microbiota^7^. Evidence exists to suggest both may in fact be interrelated and influenced by each other, for example, diet and microbiota can influence DNA methylation patterns by providing epigenetically active metabolites such as folate, butyrate and acetate^8^.

The first 3 years of life has been identified as an important period in which the gut microbiota evolves towards an adult-like state^9^ with the cessation of breastfeeding being identified as a major factor in determining the exact timing of the shift from an infant to adult style microbiota^10^,^11^. The microbiota profile typically ascribed to Western societies is characterised by an increased Firmicutes to Bacteroidetes (F:B) ratio^12^ and reduced diversity 9,12. Indeed, from a young age the gut microbiota profiles derived from Western populations clusters separately to those from non-Western societies^12^, with differences in diet thought to be a major cause^9^.

Furthermore, children also make a rapid and dramatic transition in eating behaviours and patterns during their first 2 years of life, which takes them from consuming all their nutrients as milk to consuming more complex and diverse foods^13^. For instance, the introduction of complementary feeding is associated with an increase in protein intake^14^ and the supply of a vast array of dietary glycans^15^. It is likely that both the nutritive and non-nutritive components of food and diet during this critical period influence how the microbiota develops^7^. In addition, behavioural and psycho-social factors associated with infant feeding also play a role by contributing to the formation of food preferences, eating behaviours and long term diet^2^,^16^.

To date, much of our understanding of the relationships between diet and the gut microbiota results from studies that employed extreme dietary modifications^17^,^18^. Alternatively, Wu *et al.*^19^ examined the association between habitual and recent diet with gut microbiota composition in 98 subjects aged between 2 to 50 years. Diet was described using intakes of 216 nutrients, whose relationships with microbiota composition tended to cluster within their macronutrient group, leading to broad conclusions similar to those obtained from studies exploring dietary extremes. The use of nutrient intakes to summarise dietary intake is problematic because it does not take into account the complex interplay of nutrients and non-nutrients within foods and diets^20^. It can also be a challenge to translate this type of information to practical public health messages because people eat food, meals and diets, rather than individual nutrients^21^.

Despite the widespread view that diet is one of the most important and potentially modifiable determinants of the gut microbiota, there is currently a lack of evidence in these areas that can be effectively used to support practical nutritional advice. To that end, the aim of this study was to assess the habitual and recent food intake of a group of 2 to 3 years old children and identify any associations with microbiota composition.

## Results

### Study Cohort

37 children (female = 16) aged 2.24 to 3.13 years (median = 2.61 years) were recruited between December 2012 and October 2013. Adonis was used to explore the influence of participant characteristics on microbiota composition as quantified using weighted UniFrac distances (see Supplementary Table 1).

### Food Intake

Data on habitual diet were collected using a validated food frequency questionnaire (FFQ)^22^ while data on recent dietary intake were collected using 24 hour recalls for the 3 days prior to stool sample collection. Data were converted into serve intake of food groups and subgroups (see Supplementary Table 3 and 4). The median, minimum and maximum daily serve intakes for each food group and the Spearman’s rank correlation between the 24 hour recall and FFQ data are summarised in Table 1. For all food groups, except grains, the median daily serve intakes estimated from the FFQ data were higher compared to those estimated from the 24 hour recall data. This may reflect differences between the mother’s perception of a serve size as used for the FFQ and the standard adult sized portion used by Foodworks 8 to convert the 24 hour recall data into serve intake data. Nonetheless, the serve intake data from the FFQ and 24 hour recalls were significantly correlated for the animal protein, dairy and vegetarian protein (soy, pulses and nuts) food groups.

The intakes of the fruit and vegetable food groups calculated from both methods were not significantly correlated. This may suggest that fruit and vegetable intakes are variable resulting in differences between recent and habitual intake. It is also possible that as fruits and vegetables are widely recognised as desirable components of a healthy diet there may have been an element of over reporting in the FFQ data. Numerous studies have noted an increased reported consumption of healthy foods, such as fruits and vegetables, as measured using FFQ compared to 24 hour recalls^23^,^24^,^25^ which supports this hypothesis. The maximum daily serve intake of fruit calculated using the FFQ data (12.23) was notably high. This figure was calculated by adding the reported serve intake for 6 fruits available throughout the year and 5 fruits available seasonally. The FFQ contained a separate question which asked mothers to estimate their child’s overall daily frequency of consumption of pieces of fruit in the previous 6 months. The median reported intake was 2.5 (min = 0, max = 5) pieces of fruit which appears a more reasonable estimation of fruit intake. This value correlated with the daily serve intake of fruit calculated from the FFQ (rho = 0.45; p = 0.005; n = 37) but not from the 24 hour recall data (rho = −0.24, p = 0.156; n = 37) using Spearman’s rank correlation, providing some validity to the FFQ data.

The association between the daily serve intakes of food groups calculated using the FFQ and 24 hour recall data can be found in Supplementary Table 5 and 6. For the FFQ data, fruit serve intake was positively associated with vegetable serve intake (rho = 0.51, p = 0.001) and vegetarian protein (soy, pulses and nuts) serve intake (rho = 0.38, p = 0.019), while dairy serve intake was negatively associated with vegetarian protein serve intake (rho = −0.57, p = <0.001).

### Food Group Intake and Microbiota Diversity

Spearman’s rank correlation (n = 37) was used to explore the association between food group intakes and species richness (Chao1) and diversity (Shannon Index) (Table 2). Dairy serve intake as calculated from the FFQ data was negatively associated with diversity (rho = −0.45, p = 0.006, pFDR = 0.036) while dairy serve intake calculated from the 24 hour recall data was negatively associated with richness (rho = −0.51, p = 0.001, pFDR = 0.006). Fruit and vegetarian protein (soy, pulse, nut) serve intakes calculated from the FFQ data were positively associated with diversity but after correction for FDR these associations were no longer significant.

### Food Group Intake and Microbiota Composition

The effect of food group intake on the overall faecal microbiota composition was explored by applying Adonis (n = 37) on the weighted UniFrac distances (Table 3). Dairy and vegetarian protein (soy, pulses, and nuts) serve intakes, calculated from both the FFQ and 24 hour recall data, were significant in explaining between 7 to 10% of the variation in microbiota composition. In addition, fruit serve intake calculated from the FFQ data and vegetables serve intake calculated from the 24 hour recall data were both significant in explaining 8% of the variation in microbiota composition.

To identify the particular foods associated with microbiota composition (weighted UniFrac distances) the Adonis analysis was repeated using the serve intakes of each sub-group within the dairy, vegetarian protein and fruit food groups, as calculated using the FFQ data (Table 4). Yoghurt was significant in explaining 9% of the variance in microbiota composition (p = 0.006, pFDR = 0.018) while milk alternatives (soy and rice milk) and soy products were significant (pFDR = 0.048) in explaining 6 and 7% of the variance in microbiota composition, respectively. The apple or pear sub-group and the citrus fruit sub-group intakes were associated with microbiota composition but these associations did not remain significant after correction for FDR. The same analysis undertaken, using the 24 hour recall data, revealed no significant associations after correction for FDR (Table 4).

### Food Group Intake and Relative Abundance of Taxa

For the food groups and sub groups found to be significantly associated with microbiota composition, Spearman’s rank correlation (n = 37) was used to explore the associations between serve intakes and the relative abundance of taxa at the phylum, genus, species and OTU level. Scatterplots of all reported associations can be found in Supplementary Figures 1–5.

At the phylum level (see Supplementary Figure 1), dairy serve intake (FFQ data) was negatively associated with the relative abundance of members of the Bacteriodetes phylum (rho = −0.48, p = 0.002, pFDR = 0.015), which was also reflected in a significant positive association of dairy intake with the F:B ratio (rho = 0.47, p = 0.003, pFDR = 0.018). In contrast, vegetarian protein (soy, pulses and nuts) serve intake (FFQ data) was negatively associated with the relative abundance of the phylum Firmicutes (rho = −0.44, p = 0.006, pFDR = 0.041) (see Supplementary Figure 1).

Within the Firmicutes phylum (see Supplementary Figure 2), the relative abundance of an OTU assigned to the genus *Streptococcus* was positively associated with yoghurt serve intake (FFQ data) (rho = 0.65, p < 0.001, pFDR = 0.001) and dairy serve intake (24 hour recall data) (rho = 0.58, p < 0.001, pFDR = 0.014). The reference sequence for this OTU was shown to be 100% identical to 3 reference sequences assigned to *Streptococcus salivarius* subsp. thermophilus using SINA (SILVA Incremental Aligner). In addition, dairy serve intake (24 hour recall data) was positively associated with the relative abundance of an OTU assigned to the genus *Lachnoclostridium* (rho = 0.51, p = 0.001, pFDR = 0.043) and an OTU assigned to genus *Erysipelatoclostridium* (rho = 0.51, p = 0.001, pFDR = 0.043), which was shown to be 98.3–99.9% identical to 4 reference sequences for *Erysipelatoclostridium ramosum* using SINA. Conversely, dairy serve intake (24 hour recall) was negatively associated with the relative abundance of an OTU assigned to species *Faecalibacterium prausnitzii* (rho = −0.58, p = <0.001, pFDR = 0.014), and another assigned to *Faecalicbacterium* spp. (rho = −0.55, p < 0.001, pFDR = 0.026) as well as an OTU assigned to the genus *Fusicatenibacter* (rho = −0.51, p = 0.001, pFDR = 0.043).

Within the Bacteroidetes phylum (see Supplementary Figure 3), yoghurt serve intake (FFQ data) was negatively associated with the relative abundance of the genus *Alistipes* (rho = −0.62, p ≤ 0.001, pFDR = 0.001) and *Bacteroides* (rho = −0.506, p = 0.001, pFDR = 0.014). Similarly, dairy serve intake (24 hour recall) was negatively associated with the relative abundance of unspecified species of the genus *Parabacteroides* (rho = −0.51, p = 0.001, pFDR = 0.027) and an OTU assigned to the genus *Bacteroides*, which was 99.86% identical to reference sequences for *Bacteroides faecis* on SINA. In contrast, vegetarian protein serve intake (24 hour recall data) was positively associated with the relative abundance of an OTU assigned to the genus *Bacteroides* (rho = 0.58, p < 0.001, pFDR = 0.031), which was shown to be 99.85% identical to reference sequences for *Bacteroides xylanisolvens* using SINA.

Vegetable serve intake (24 hour recall data) was positively associated with the relative abundance of the *Lachnospira* genus (rho = 0.49, p = 0.002, pFDR = 0.032) and negatively associated with an unspecified genus of the order Clostridiales (rho = −0.51, p = 0.001, pFDR = 0.032) (see Supplementary Figure 4).

Fruit serve intake (FFQ data) was negatively associated with the F:B ratio (rho = −0.40, p = 0.013, pFDR = 0.039), the relative abundance of unspecified genus of the family Erysipelotrichaceae (rho = −0.51, p = 0.001, pFDR = 0.020), and species related to *Ruminococcus gnavus* (rho = −0.54, p = <0.001, pFDR = 0.022). No significant associations were observed between serve intakes of the citrus sub –group and any taxa though the apple/pear sub-group was negatively associated with relative abundance of *Ruminococcus gnavus* (rho = −0.56, p ≤ 0.001, pFDR = 0.012) (see Supplementary Figure 5).

### Food Group Intake and Microbial Metabolic Pathways

Spearman’s rank correlation test (n = 37) was used to explore the association between food group intakes and the abundance of KEGG functional pathways. Total fruit intake (FFQ data) was positively associated with the digestive system level 2 KEGG functional pathway (rho = 0.58, p < 0.001, pFDR = 0.005) and specifically the protein digestion and absorption level 3 KEGG functional pathway (rho = 0.46, p = 0.003, pFDR = 0.026).

## Discussion

Dairy and vegetarian protein (soy, pulses and nuts) serve intakes were significantly associated with microbiota composition while being negatively correlated with each other. The FFQ data revealed that the relative abundance of Bacteroidetes was negatively associated with dairy serve intake while the relative abundance of Firmicutes was negatively associated with vegetarian protein serve intake. Dairy serve intake was also negatively associated with measures of diversity. This supports the findings of Butteiger *et al.*^26^ who found that feeding hamsters soy protein, as opposed to milk protein, resulted in a significant impact on microbiota composition and an increase in microbial diversity. Notably, a positive association was observed between vegetarian protein serve intakes (soy, pulse and nuts) and the relative abundance of an OTU related to *Bacteroides xylanisolvens,* which unlike most other *Bacteroides* species is unable to degrade starch^27^. Although all legumes would provide a source of xylan and related polysaccharides, soybeans are unique among legumes in that they contain very little starch^28^, perhaps suggesting that soy intake was the primary source of nutrients driving the increase in the abundance of *Bacteroides xylanisolvens*.

The gut microbiota of non-Western individuals from hunter-gatherer and agricultural societies are reported to possess greater diversity, leading to reduced F:B ratios compared to individuals from Western societies^12^,^29^,^30^. These differences are often attributed to a reduction in bacterial diversity within the Western diet and environment^31^. Another distinguishing feature of the Western diet is the intake of significant quantities of dairy and particularly pasteurised milk products, which would provide a source of high quality protein but a relatively “sterile” microbiota composition, limited only to those strains used for the production of fermented milk products, such as yoghurt.

Dairy serve intake, and more specifically yoghurt serve intake, were positively associated with the relative abundance of an OTU related to *Streptococcus salivarious* ssp. thermophilus (see Supplementary Figure 2). *S. salivarious* ssp. thermophilus is a commonly used starter culture for milk fermentation in yoghurt production, though previous studies have suggested a low survival rate through the gastro-intestinal tract^32^. Alvaro *et al.*^33^ found a significant correlation between faecal β-galactosidase activity and yoghurt consumption and noted that *S. salivarious* ssp. thermophilus contains β-galactosidase, suggesting an important metabolic role for this bacterium. The β-galactosidase enzyme performs the same function as lactase and could be responsible for the link between yoghurt consumption and improved lactose digestion in individuals with lactose intolerance^34^. Conversely, yoghurt serve intake was found to be negatively associated with the relative abundance of *Bacteroides*. Both fresh and heat treated yoghurt have been associated with a decrease in faecal *Bacteroides*^35^ suggesting this association is not related to the live cultures found within yoghurt.

The dairy food group serve intake was also positively associated with the relative abundance of OTUs with high similarity to *Lachnoclostridium spp.* and *Erysipelatoclostridium ramosum. E. ramosum* has been linked to metabolic syndrome in humans; and by animal studies, associated with upregulation of small intestinal glucose and fat transporters resulting in enhanced diet-induced obesity^36^. Further evidence for the link between early life dairy consumption and obesity is provided by Gunther at al^37^ who found that increased protein intake from dairy, but not meat or cereals, at 12 months was associated with increased BMI (Body Mass Index) and body fatness at age 7 years. In contrast, consumption of dairy products by older children and adults is often considered to protect against overweight and obesity although the current body of evidence is neither consistent nor conclusive^34^,^38^.

Fruit serve intake (FFQ data) and vegetable serve intake (24 hour recall data) were significantly associated with microbiota composition. De Filippis *et al.*^39^ reported that at the genus level *Lachnospira* and *Prevotella* were positively associated with plant based diets while *Ruminococcus* and *Streptococcus* correlated positively with nutrients of animal origin and negatively with a vegetable-based dietary pattern. This directly supports the results from this study in that vegetable serve intake was positively associated with the relative abundance of *Lachnospira*, fruit serve intake was negatively associated with the relative abundance of *Ruminococcus* and dairy serve intake was positively associated with the relative abundance of *Streptococcus*. In the first 100 days of life the relative abundance of *Lachnospira* has been shown to be transiently reduced in the microbiota of children later identified as being at risk of developing asthma^40^. It is well established that higher vegetable intakes in mothers’ diets during pregnancy^41^ and the child’s own diet^42^ are associated with a reduced risk of asthma in children. Our findings are consistent with there being a link between vegetable intake, *Lachnospira* abundance and asthma risk.

Our finding that total fruit and apple/pear serve intakes were both shown to be negatively associated with the relative abundance of species related to *Ruminococcus gnavus* is somewhat surprising. Some strains of this bacterium have the capacity for growth on host-derived mucins in addition to dietary sources of carbohydrates^43^ while their absolute abundance is favoured by a diet with a relatively high FODMAP content^44^. As such, the negative associations observed here do not appear to be directly related to the carbohydrate content of these foods, but perhaps, are more of a reflection of the competition for niche occupation, and that these foods favour the growth and abundance of other unidentified bacterial groups, reflected in a reduced relative abundance of *R. gnavus* in these compositional profiles. Further support for this association is provided by a recent large Dutch population based study which found that fruit intake was negatively associated with the abundance of the closely related *R. torques* in a cohort of 1179 adults^45^. This study identified chromogranin A (CgA) as being strongly negatively associated with microbiota composition, microbial diversity and functional gene richness. CgA is a member of the granine peptides, which are secreted in nervous, endocrine, and immune cells under stress and during active periods of gut –related diseases such as Irritable Bowel Syndrome and Inflammatory Bowel Disease. Interestingly, CgA was negatively correlated with fruit and vegetable intake and positively correlated with the abundance of both *R. gnavus* and *torque,* which is consistent with *R. gnavus* having been shown to be enriched in the microbiota of humans with Inflammatory Bowel Disease^43^. In addition, this study found that fruit intake was positively associated with the MetaCyc functional pathway for lysine fermentation to acetate and butyrate (P163_PWY) and negatively with the lysine biosynthesis (PWY_2941) pathway which is consistent with our finding that total fruit intake was positively correlated with the protein digestion and absorption level 3 KEGG functional pathway. Recent studies suggest that *Ruminococcus gnavus* is important for the “maturation” of the gut microbiota^10^ and supports the reversal of growth impairments observed in germ-free mice colonised with the faecal microbiota derived from undernourished children^46^. The colonised mice’s metabolic phenotypes suggested a diversion of amino acids away from oxidation and towards protein synthesis and lean mass formation^46^, which suggests *R. gnavus* is able to interact with host protein metabolism. Further studies are required to understand the relationships between fruit intake, *Ruminococcus* spp, gut health and microbial and host protein metabolism.

This study revealed associations between dairy and plant- based foods (fruit, vegetables, soy, pulses and nuts) and microbiota composition in young children. Individual foods are not consumed in isolation but rather within a varied and variable diet. In this study fruit serve intake was positively correlated with vegetable serve intake and vegetarian protein (soy, pulses and nuts) serve intake, while dairy serve intake was negatively correlated with vegetarian protein serve intake. This study warrants repeating in a larger cohort to better appreciate the interrelationships of foods as they are consumed and the synergistic effects of these dietary patterns on the microbiota. Despite a small sample size and associated limitation to the power to identify statistically significant associations the use of food rather than nutrient intakes revealed numerous food - microbiota associations. Further exploration of these has the potential to suggest effective dietary interventions to prevent microbiota dysbiosis and associated disease.

## Method

### Study participants

Thirty seven children aged 2 to 3 years were recruited from the ongoing Feeding Queensland Babies Study (FQBS) cohort^47^ between December 2012 and October 2013. Exclusion criteria included: pre-existing gastrointestinal and immunodeficiency disease; antibiotic use in the previous 3 months; medications known to impact microbiota in the previous 4 weeks; and NSAIDS or antacids in the previous 2 weeks. Weight and height were assessed at the study visit and used to calculate weight for age, height for age and BMI for age Z scores using WHO reference data^48^.

### Diet analysis

Data on habitual diet were collected using a validated food frequency questionnaire (FFQ)^22^ which asked mothers to report their child’s usual serve intake of 120 items over the past 6 months. These data were combined into daily serve intakes of 6 food groups and 27 subgroups (see Supplementary Table 2). Data on recent dietary intake were collected using 24 hour recalls for the 3 days prior to stool sample collection. The 24 hour recalls were administered by an Accredited Practising Dietitian (Dietetic Association of Australia) and mothers were asked to estimate quantities consumed using household measures. Foodworks 8 (Xyris Software, Australia) was used to convert data from the 24 hour recalls into daily serve intakes of food groups and subgroups using standard adult sized serves (see Supplementary Table 3). Foodworks 8 includes legumes in both the vegetable and protein foods food groups and includes milk alternatives within the dairy food group. To make the food groups mutually exclusive and to provide comparability with the FFQ food groups a vegetarian protein food group was manually created to include the sub-groups for nuts and seeds, legumes, soy products, and milk alternatives, resulting in a total of 6 food groups and 26 subgroups calculated from the 24 hr recall data.

### Microbiota

Faecal samples were collected from a disposable bed pan (or nappy if not toilet trained) at the participant’s homes within 24 hours of the study visit and frozen immediately at −20 C. The frozen samples were transported in insulated bags with frozen ice blocks before being transferred to −80 C for storage. Faecal DNA extraction, PCR amplification and library construction for bar-coded 16S rRNA gene amplicon sequencing, using the Illumina Mi-Seq platform, was performed following standard operating protocols used by the Australian Centre for Ecogenomics, University of Queensland, Australia (ecogenomic.org). Detailed DNA extraction and sequencing methods are provided in Supplementary Information.

### Bioinformatics

QIIME 1.9.0^49^ was used for bioinformatics. QIIME’s pick_open_reference_otus.py workflow was used to generate OTUs using default parameters (97% sequence similarity; Greengenes reference database–version 13 8^50^ ; uclust OTU picking method^51^). The resulting OTU table was filtered to remove any OTU with a relative abundance of less than 0.05% across all samples. OTUs of significance that were not initially taxonomically classified were aligned with reference sequences using SINA (SILVA Incremental Aligner)^52^ to provide further identification.

Microbiota composition was described using α and β diversity measures. α-diversity refers to the variety and abundance of species within a sample while β-diversity refers to the difference in α-diversity between samples^53^. α-diversity can be described using richness, which reflects the number of species within a sample and evenness, which measures the similarity in abundance of species. Species richness was estimated using Chao1^54^ while Shannon Index was used to estimate diversity, reflecting both richness and evenness. β-diversity was calculated using the weighted UniFrac distance metric^55^ which is a phylogenetic distance measure that quantifies the distance between communities based on the lineages they contain. The OTU table was rarefied to the minimum sample count (42629 reads) for calculation of measures of diversity to control for sequencing depth. Relative counts (read count divided by total reads for that sample) at phylum, genus, and species level were created using the summarize_taxa.py script in QIIME. Firmicutes –Bacteroidetes (F-B) ratio was calculated by dividing log abundances using the compute_taxonomy_ratio.py script in QIIME.

### Statistics

Adonis^56^ was employed in QIIME to explore associations between with food intakes and the weighted UniFrac distance metric. PICRUSt 1.0.0^57^ was used on the online Galaxy interface (http://huttenhower.sph.harvard.edu/galaxy/) to predict KEGG functional pathways at Level 2 and 3 using a closed reference OTU table created in QIIME using the filter_otus_from_otu_table.py script and the Greengenes reference database–version 13 8^50^. Spearman’s rank correlation was used to explore the impact of food intake on α –diversity and taxa and KEGG functional pathway abundance. P values were adjusted for multiple testing using the False Discovery Rate (FDR) Benjamini-Hochberg procedure^58^.

### Ethics

This study was approved by The University of Queensland Medical Research Ethics Committee (Approval Number: 2012001155) and the Metro South Hospital and Health Service Human Research Ethics Committee (HREC Ref: HREC/12/QPAH457) in Brisbane, Australia and conducted in accordance with the principles expressed in the Declaration of Helsinki. All participants were provide with written and verbal information and consent forms were signed by the mother or a legal guardian.

## Additional Information

**Accession codes**: All raw data and related metadata underlying the findings reported in this manuscript are deposited in the Qiita (qiita.ucsd.edu) public repository (EBI accession number ERP016652).

**How to cite this article**: Smith-Brown, P. *et al.* Dairy and plant based food intakes are associated with altered faecal microbiota in 2 to 3 year old Australian children. *Sci. Rep.* **6**, 32385; doi: 10.1038/srep32385 (2016).

## Supplementary Material

Supplementary Information

## Figures and Tables

**Table 1:** Daily serve intake of food groups as calculated using the 24 Hour Recall and FFQ data and Spearman’s rank correlation (n = 37) between them.

Food Group	24 Hr Recall median (min-max)	FFQ median (min-max)	Spearman Correlation	
			rho	p
Grains	4.63 (1.58–10.15)	3.07 (1.29–5.08)	0.11	0.515
Fruit	1.58 (0.34–4.06)	3.05 (0.02–12.23)	−0.20	0.225
Vegetables	1.35 (0.30–4.05)	3.57 (1.23–8.53)	0.31	0.062
Animal protein	0.56 (0.00–1.53)	1.88 (0.09–4.34)	0.48*	0.002
Vegetarian protein	0.18 (0.00–0.99)	0.66 (0.02–3.07)	0.33*	0.043
Dairy	1.94 (0.01–3.82)	4.07 (0.18–7.00)	0.63*	<0.001

^*^p < 0.05.

**Table 2:** Results of Spearman rank correlation (n = 37) between food group intake (as calculated using the 24 hour recall and FFQ data) and diversity (Shannon Index) and richness (Chao1) of microbiota.

Food Group	24 Hr Recall						FFQ					
	Diversity (Shannon Index)			Richness (Chao1)			Diversity (Shannon Index)			Richness (Chao1)		
	rho	p	pFDR	rho	p	pFDR	rho	p	pFDR	rho	p	pFDR
Grains	−0.29	0.084	0.231	0.00	0.987	0.987	−0.04	0.803	0.803	0.01	0.951	0.951
Fruit	0.24	0.151	0.231	0.16	0.334	0.6915	0.36	0.029	0.062	0.23	0.170	0.51
Vegetables	0.24	0.154	0.231	−0.13	0.432	0.6915	0.11	0.507	0.7605	0.14	0.409	0.6135
Animal protein	−0.13	0.450	0.450	−0.02	0.897	0.987	−0.071	0.676	0.803	−0.08	0.650	0.78
Vegetarian protein	0.18	0.291	0.349	−0.125	0.461	0.6915	0.355	0.031	0.062	0.17	0.314	0.6135
Dairy	−0.36	0.031	0.186	−0.51*	0.001	0.006	−0.45*	0.006	0.036	−0.28	0.091	0.51

^*^pFDR < 0.05.

**Table 3:** Results of Adonis analysis (n = 37) of association between food group intake (as calculated using the 24 hour recall and FFQ data) and microbiota composition (weighted UniFrac distance metric).

Food Group	24 Hr Recall			FFQ		
	R^2^	p	pFDR	R^2^	p	pFDR
Grains	0.03	0.366	0.439	0.02	0.536	0.804
Fruit	0.04	0.207	0.310	0.08*	0.015	0.030
Vegetables	0.08*	0.014	0.042	0.02	0.699	0.839
Animal protein	0.015	0.811	0.811	0.01	0.963	0.963
Vegetarian protein	0.10*	0.005	0.030	0.08*	0.009	0.027
Dairy	0.07*	0.024	0.048	0.10*	0.006	0.027

^*^pFDR < 0.05.

**Table 4:** Results of Adonis analysis (n = 37) of association between sub-group intake (as calculated using the 24 hour recall and FFQ data) and microbiota composition (weighted UniFrac distance metric).

		24 Hr Recall FFQ			FFQ		
Food Group	Sub-Group	R^2^	p	pFDR	R^2^	p	pFDR
Fruit	Apple or pear				0.06	0.042	0.105
	Orange, mandarin, grapefruit				0.08	0.010	0.050
	Banana				0.01	0.958	0.958
	Seasonal fruit				0.05	0.071	0.118
	Mixed fruit				0.03	0.434	0.542
Vegetables	Dark green	0.04	0.208	0.277			
	Red orange	0.05	0.085	0.277			
	Starchy	0.03	0.364	0.364			
	Other	0.04	0.159	0.277			
Vegetarian protein	Milk alternatives	0.05	0.051	0.196	0.06*	0.024	0.048
	Soy products	0.04	0.229	0.305	0.07	0.017	0.048
	Legumes	0.05	0.098	0.196	0.04	0.185	0.247
	Nuts and seeds	0.02	0.498	0.498	0.03	0.416	0.416
Dairy	Milk	0.03	0.409	0.409	0.04	0.172	0.172
	Cheese	0.04	0.182	0.273	0.05	0.099	0.148
	Yoghurt	0.05	0.061	0.183	0.09*	0.006	0.018

^*^pFDR < 0.05.
