# Supplementary material for: Dairy and plant based food intakes are associated with altered faecal microbiota in 2 to 3 year old Australian children
Source: Sci Rep. 2016 Oct 3;6:32385. doi: 10.1038/srep32385 (PMC5046176; doi:10.1038/srep32385)

SUPPLEMENTARY INFORMATION

Dairy and plant based food intakes are associated with altered faecal microbiota in 2 to 3 year old Australian children.

P Smith-Brown, M Morrison, L Krause, PSW Davies

DNA extraction and sequencing methods 3

Supplementary Tables

Supplementary Table 1: Study participant characteristics and association with microbiota composition (weighted UniFrac distance metric) as calculated using Adonis. 6

Supplementary Table 2: Daily serve intake of 6 food groups and 27 sub-groups as calculated using the FFQ data. 7

Supplementary Table 3: Daily serve intake of 6 food groups and 26 sub-groups as calculated using the 24 hour recall data 8

Supplementary Table 4: Spearman rank correlation between daily serve intake of food groups as calculated from the FFQ data. 9

Supplementary Table 5: Spearman rank correlation between daily serve intake of food groups as calculated from the 24 hour recall data. 10

Supplementary Figures:

Supplementary Figure 1: Associations between dairy intake and relative abundance of Bacteroidetes and vegetarian protein intake and relative abundance of Firmicutes. 11

Supplementary Figure 2: Associations between dairy and yoghurt intake and relative abundance of OTU within Firmicutes phylum. 12

Supplementary Figure 3: Associations between dairy, yoghurt and vegetarian protein (soy, pulses and nuts) intakes and relative abundance of taxa within the Bacteroidetes phylum. 13

Supplementary Figure 4: Associations between vegetable intake and relative abundance of taxa at genus level. 14

Supplementary Figure 5: Association between fruit intake and relative abundance of taxa at genus and species level. **.. 15**

DNA Extraction and Sequencing Methods

DNA Extraction

DNA was extracted from 50-100 mg of raw sample which was weighed and recorded. An initial step of bead beating using 0.7mm Garnet Bead Tubes (Mo-Bio #13123-50) was performed as per the manufacturer's instructions. Sample was added into a bead tube filled with 750 μl of Tissue Lysis Buffer (Promega #A5091). Tubes were vortexed using the Vortex Genie (Mo-Bio #13111, tube adapter #13000-V1-24) for 10 mins at max speed and subsequently centrifuged at 10,000g for 30 seconds (Hereaus Pico). 300 µl of the resulting lysate was added into each well of the Promega Maxwell 16 cartridge. DNA extraction was performed using the Maxwell® 16 Research Instrument (Promega) according to the manufacturer's protocol with the Maxwell 16 Tissue DNA Purification Kit (Promega Cat. #AS1030). DNA was eluted using the SEV setting which is the standard elution volume as outlined in the manufacturer's instructions. DNA concentration was measured using a Qubit broad range assay (ThermoFisher Scientific; Qubit 3.0 and #Q32853) and was adjusted to a concentration of 5ng/ul.

A quantitative polymerase chain reaction (qPCR) analysis was performed to quantify microbial load and check for inhibitors in the extracted DNA, as follows. The PCR was set up using 5µl of 2X SYBR Green/AmpliTaq Gold DNA Polymerase mix (Life Technologies, Applied Biosystems), 4µl of microbial template DNA and 1µl of primer mix. The 16S 1406F/1525R primer set (0.4µM) was designed to amplify bacterial and archaeal 16S rRNA genes: F - GYACWCACCGCCCGT and R - AAGGAGGTGWTCCARCC. The rpsL F/R primer set (0.2µM), used for inhibition control, amplifies Escherichia coli DH10B only: F - GTAAAGTATGCCGTGTTCGT and R - AGCCTGCTTACGGTCTTTA. Three dilutions 1/100, 1/500

and 1/1000 (microbial template DNA, 16S 1406F/1525R primer set) as well as an inhibition control (E. coli DH10B genomic DNA, rpsL primer set) were run in triplicate for each sample. The PCR was run on the ViiA7 platform (Applied Biosystems) including a cycle of 10 min at 95C (AmpliTaq activation) and 40 cycles of [15s at 95C followed by 20s at 55C and 30 s at 72C]. A melt curve was produced by running a cycle of 2 min at 95C and a last cycle of 15s at 60C. The cycle threshold (Ct) values were recorded and analyzed using ViiA7 v1.2 software. The extracted DNA was determined to be of sufficient quality to proceed without the need for any further dilution or clean up protocols.

PCR amplification and Amplicon sequencing

The 16S rRNA gene encompassing the V6 to V8 regions was targeted using the 926F (5'-AAACTYAAAKGAATTGRCGG-3') and 1392R (5'-ACGGGCGGTGWGTRC-3') primers [1] modified to contain Illumina specific adapter sequence (926F: 5'TCGTCGGCAGCGTCAGATGTGTATAAGAGACAGAAACTYAAAKGAATTGRCGG3'and 1392wR:5'GTCTCGTGGGCTCGGGTCTCGTGGGCTCGGAGATGTGTATAAGAGACAGACGGGCGGTGWGTRC3'). The universal primer pair Univ_SSU_926F-1392wR amplifies the small subunit (SSU) ribosomal RNA of eukaryotes (18S) and prokaryotes (16S) specifically the V6, V7 and V8 regions. In *Escherichia coli*, it amplifies the 926-1392 region of the 16S gene.

Preparation of the 16S library was performed as described, using the workflow outlined by Illumina (#15044223 Rev.B). In the 1st stage, PCR products of ~466bp were amplified according to the specified workflow with an alteration in polymerase used to substitute Q5 Hot Start High-Fidelity 2X Master Mix (New England Biolabs) in standard PCR conditions. Resulting PCR amplicons were purified using Agencourt AMPure XP beads (Beckman

Coulter). Purified DNA was indexed with unique 8bp barcodes using the Illumina Nextera XT 384 sample Index Kit A-D (Illumina FC-131-1002) in standard PCR conditions with Q5 Hot Start High-Fidelity 2X Master Mix. Indexed amplicons were pooled together in equimolar concentrations and sequenced on MiSeq Sequencing System (Illumina) using paired end sequencing with V3 300bp chemistry in the Australian Centre for Ecogenomics according to manufacturer's protocol.

Quality Control

After trimming the first 20 bases to remove primer sequences poor quality sequences were removed using a sliding window of 4 bases with an average base quality of 15 using the software Trimmomatic. All reads were then hard trimmed to 250 bases with any less than 250 bases excluded.

Reference

1 Engelbrektson, A. *et al.* Experimental factors affecting PCR-based estimates of microbial species richness and evenness. *Isme j* **4**, 642-647, doi:10.1038/ismej.2009.153 (2010).

Supplementary Table 1: Study participant characteristics and association with microbiota composition (weighted UniFrac distance metric) as calculated using Adonis (n=37).

**Participant Characteristics**		Microbiota Composition (Weighted UniFrac)	
		R^2	p
Age	Median= 2.61 yrs<br>Min = 2.24; Max = 3.13 yrs	0.02	0.582
Gender	Female= 16	0.03	0.366
Weight for age WHO Z Score	Median = 0.27<br>Min = -1.27; Max = 1.97	0.02	0.465
Height for age WHO Z Score	Median = -0.33<br>Min = -1.31; Max = 2.86	0.005	0.995
BMI for age WHO Z Score	Median = 0.45<br>Min = -1.30; Max = 2.07	0.05	0.115
Gestation	Median= 281 days<br>Min = 196; Max = 294	0.04	0.254
Delivery mode	Vaginal= 20<br>Emergency Caesarean= 11<br>Planned Caesarean= 2<br>Missing data = 4	0.05	0.950
Feeding method at 4 months	Exclusive Breastfed= 27<br>Mixed feed= 7<br>Formula fed= 2<br>Missing data = 1	0.08	0.457
Mother's education	College= 12<br>Degree= 25	0.06	0.054
Residence	Urban= 2<br>Suburban= 33<br>Rural= 2	0.05	0.490
Sibling	Yes= 24	0.02	0.818
Pets	None= 10<br>Cat/dog= 23<br>Fish/worms= 1<br>Chicken= 3	0.02	0.552
Smoking exposure	During pregnancy & breastfeeding or in home = 2	0.02	0.538
Medical condition	Asthma/eczema/allergy= 11	0.03	0.362

*p<0.05

Supplementary Table 2: Daily serve intake of 6 food groups and 27 sub-groups as calculated using the FFQ data.

Food Group	Subgroup	Daily Serve Intake			
		**Median**	**Range**	**Minimum**	**Maximum**
Grains		3.07	3.79	1.29	5.08
	Breakfast cereal	0.86	1.72	0.00	1.72
	Bread	0.88	2.62	.02	2.64
	Noodle and pasta	0.45	.79	.14	.93
	Rice	0.14	.84	.02	.86
	Other grains	0.02	.14	0.00	.14
Fruit		3.05	12.21	.02	12.23
	Apple or pear	0.43	3.00	0.00	3.00
	Orange, mandarin, grapefruit	0.43	3.00	0.00	3.00
	Banana	0.43	3.00	0.00	3.00
	Seasonal fruit	0.74	2.37	0.00	2.37
	Mixed fruit	0.50	1.70	.02	1.72
Vegetables		3.57	7.30	1.23	8.53
	Orange veg (pumpkin, sweet potato, carrot, corn)	1.02	3.47	.11	3.58
	Red veg (capsicum, tomatoes)	0.21	1.72	0.00	1.72
	White veg (potato, cauliflower, onion, leak, mushrooms)	0.71	1.63	.16	1.79
	Green veg (green beans, spinach, cabbage, brussel sprouts, peas, broccoli, zucchini, lettuce, celery, cucumber, avocado)	1.28	3.50	.16	3.66
Animal Protein		1.88	4.25	0.09	4.34
	Chicken	0.42	1.00	0.00	1.00
	Lamb or beef	0.37	1.07	0.00	1.07
	Pork	0.04	.35	0.00	.35
	Mince meat	0.28	.57	0.00	.57
	Processed meat (sausage, bacon and luncheon meat)	0.30	1.27	.04	1.31
	Fish and seafood	0.23	.98	.02	1.00
	Eggs	0.14	.43	0.00	.43
Vegetarian protein		0.66	3.05	0.02	3.07
	Total soy (products and milk)	0.00	2.50	0.00	2.50
	Pulses	0.14	1.29	0.00	1.29
	Nuts	0.07	.86	0.00	.86
Dairy		4.07	6.82	0.18	7.00
	Dairy milk	1.57	5.00	0.00	5.00
	Yoghurt	0.57	2.98	0.02	3.00
	Cheese	1.00	3.02	0.07	3.09

Supplementary Table 3: Daily serve intake of 6 food groups and 26 sub-groups as calculated using the 24 hour recall data.

Food Group	Subgroup	Daily Serve Intake			
		**Median**	**Range**	**Minimum**	**Maximum**
Grains		4.63	8.57	1.58	10.15
	Refined grains	2.76	5.73	0.78	6.51
	Whole grains	1.86	8.05	0.04	8.08
Fruit		1.58	3.72	0.34	4.06
	Citrus, melons and berries	0.09	0.93	0.00	0.93
	Other fruit	1.24	3.18	0.14	3.31
	Fruit juice serve	0.00	1.85	0.00	1.85
Vegetables		1.35	3.75	0.30	4.05
	Dark green vegetables	0.03	0.74	0.00	0.74
	Red orange vegetables	0.35	1.51	0.03	1.54
	Tomatoes serve	0.13	0.75	0.00	0.75
	Other red orange vegetables	0.09	0.98	0.00	0.98
	Starchy vegetables	0.35	2.22	0.00	2.22
	Potatoes	0.25	2.17	0.00	2.17
	Other starchy vegetables	0.00	0.53	0.00	0.53
	Other vegetables	0.33	1.38	0.00	1.38
Animal protein		0.56	1.53	0.00	1.53
	Red meats	0.06	1.10	0.00	1.10
	Poultry	0.06	0.62	0.00	0.62
	Eggs	0.04	0.41	0.00	0.41
	Processed meats	0.08	1.03	0.00	1.03
	Seafood high in LC *n*-3 fatty acids	0.00	0.41	0.00	0.41
	Seafood low in LC *n*-3 fatty acids	0.00	0.32	0.00	0.32
Vegetarian protein		0.18	0.99	0.00	0.99
	Nuts and seeds	0.04	0.46	0.00	0.46
	Legumes (includes whole soybeans)	0.00	0.44	0.00	0.44
	Soy products (excludes soy milk and whole soybeans)	0.00	0.53	0.00	0.53
	Milk alternatives (beverages with at least 100mg calcium per 100g)	0.00	0.67	0.00	0.67
Dairy		1.94	3.82	0.01	3.82
	Dairy Milk	0.93	3.20	0.01	3.21
	Cheese	0.49	1.29	0.00	1.29
	Yoghurt	0.33	2.47	0.00	2.47

Supplementary Table 4: Spearman rank correlation (n=37) between daily serve intake of food groups as calculated from the FFQ data.

	Grains		Fruit		Vegetables		Animal protein		Vegetarian protein	
	rho	p	rho	p	rho	p	rho	p	rho	p
**Fruit**	0.24	.150								
**Vegetables**	0.03	.861	0.51*	.001						
**Animal protein**	-0.02	.885	0.15	.380	0.07	.671				
**Vegetarian protein**	0.08	.659	0.38*	.019	0.26	.127	-0.13	.443		
**Dairy**	-0.06	.703	-0.18	.292	0.09	.615	0.26	.116	-0.57*	< 0.001

*p<0.05

Supplementary Table 5: Spearman rank correlation (N=37) between daily serve intake of food groups as calculated from the 24 hour recall data.

	Grains		Fruit		Vegetables		Animal protein		Vegetarian protein	
	**rho**	**p**	**rho**	**p**	**rho**	**p**	**rho**	**p**	**rho**	**p**
**Fruit**	0.10	.570								
**Vegetables**	0.04	.815	-0.03	.846						
**Animal protein**	0.11	.518	0.02	.920	0.13	.430				
**Vegetarian protein**	-0.30	.072	-0.09	.608	0.16	.356	-0.17	.321		
**Dairy**	0.04	.798	-0.06	.711	0.09	.595	0.06	.704	-0.27	.107

Supplementary Figure 1: Associations between dairy intake and relative abundance of Bacteroidetes and vegetarian protein (soy, pulse and nut) intake and relative abundance of Firmicutes. Relative abundance data presented as log2 (relative abundance + 0.001).

a

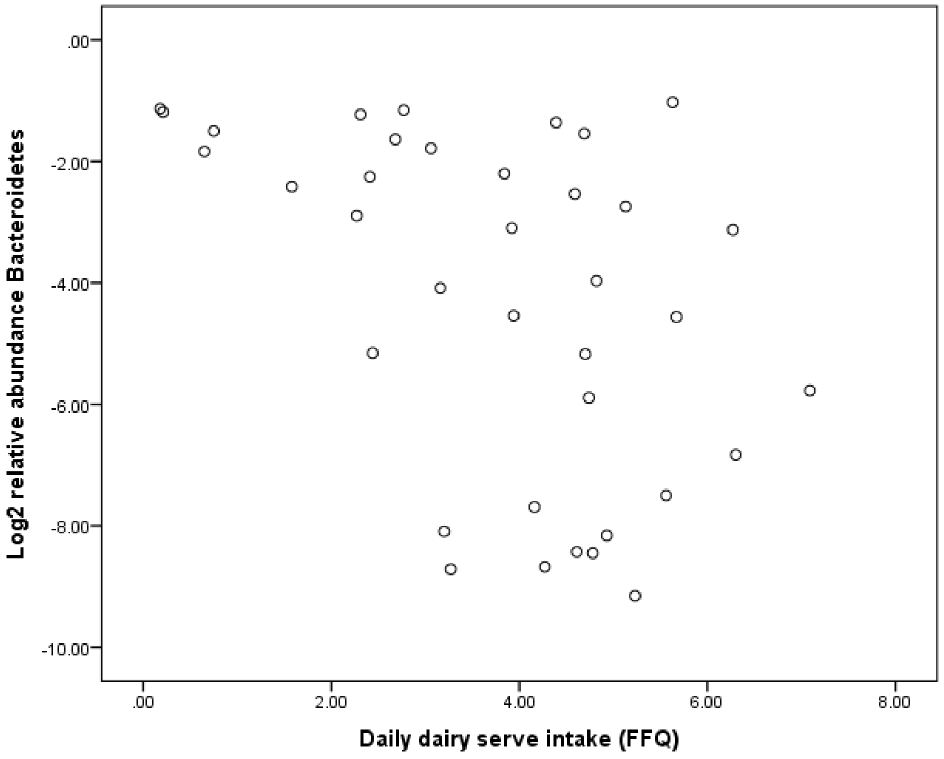

b

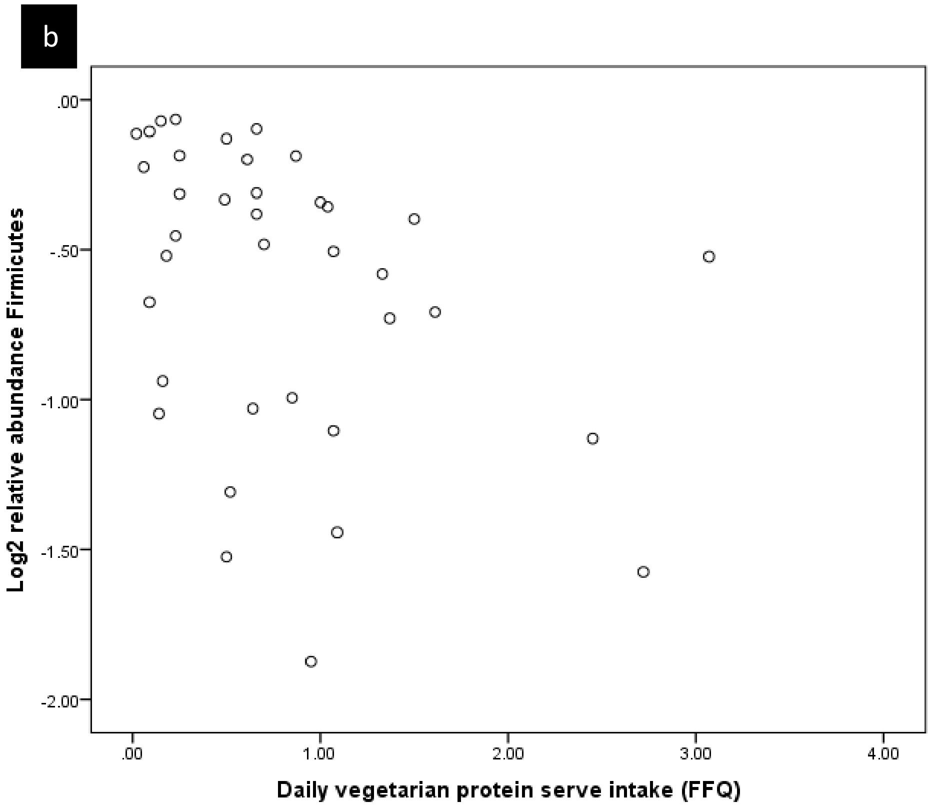

Supplementary Figure 2: Associations between dairy and yoghurt intake and relative abundance (log2 (relative abundance + 0.001)) of OTUs within Firmicutes phylum.

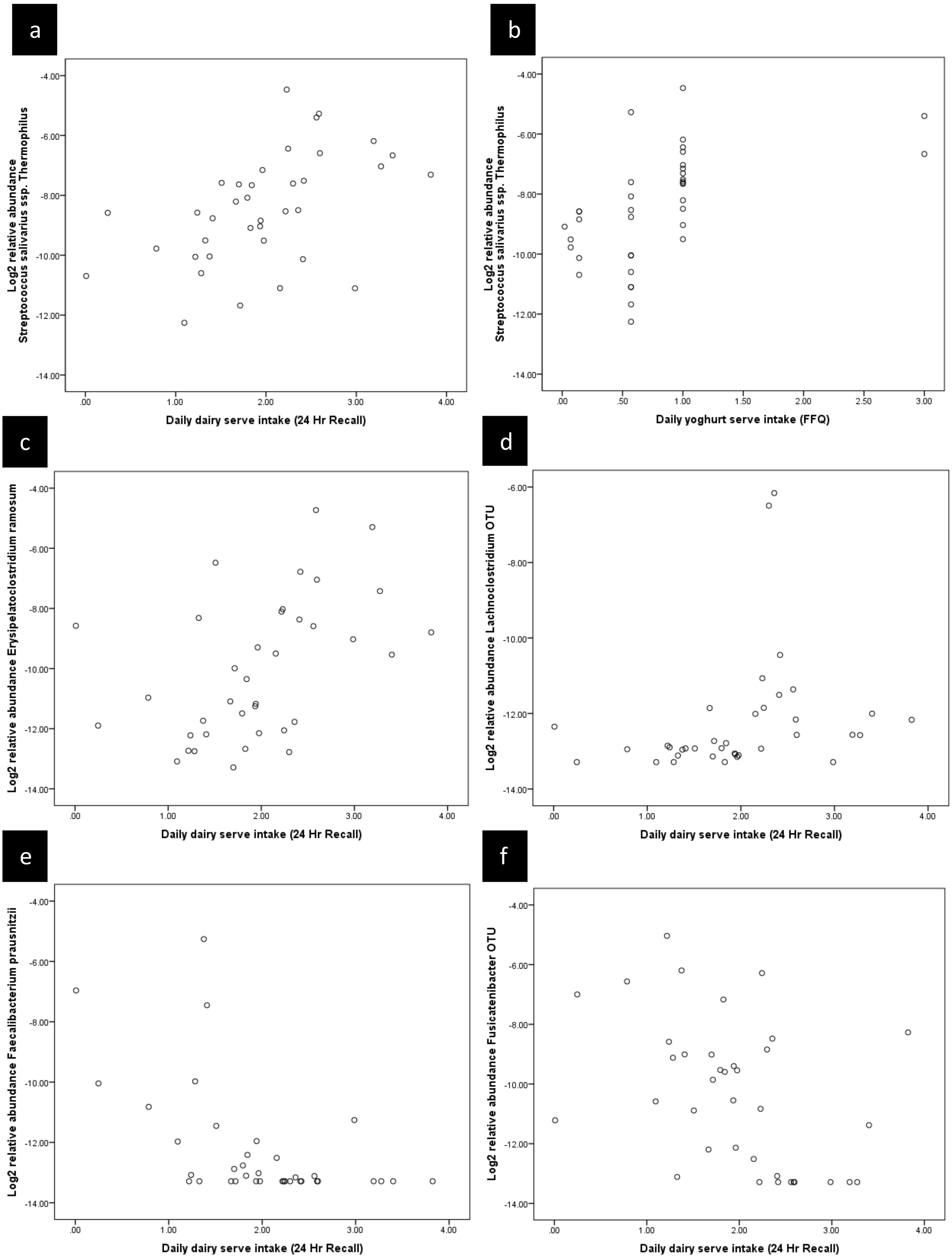

Supplementary Figure 3: Associations between dairy, yoghurt and vegetarian protein (soy, pulses and nuts) intakes and relative abundance (log2 (relative abundance + 0.001)) of taxa (genus and OTU level) within the Bacteroidetes phylum.

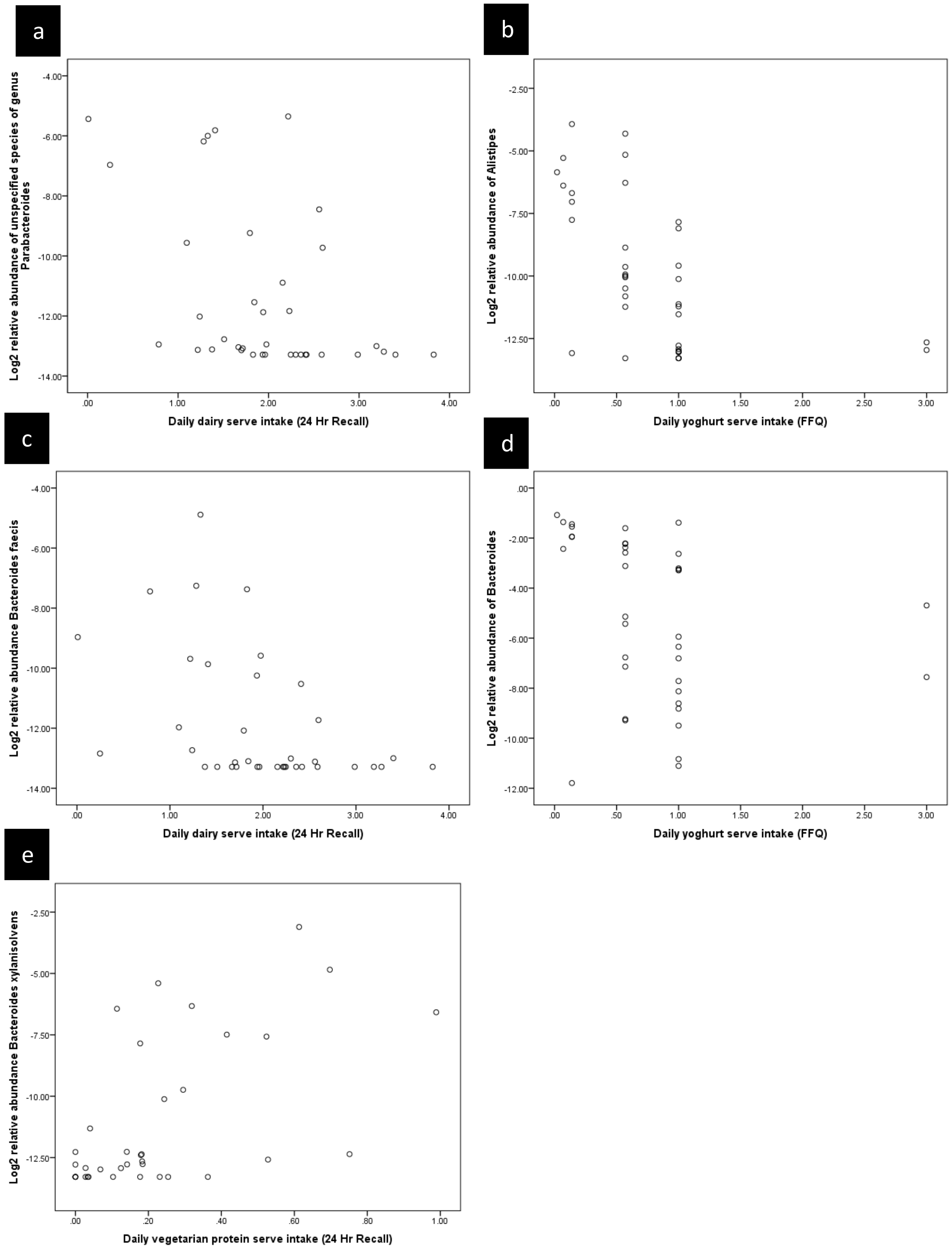

Supplementary Figure 4: Associations between vegetable intake and relative abundance (log2 (relative abundance + 0.001)) at genus level.

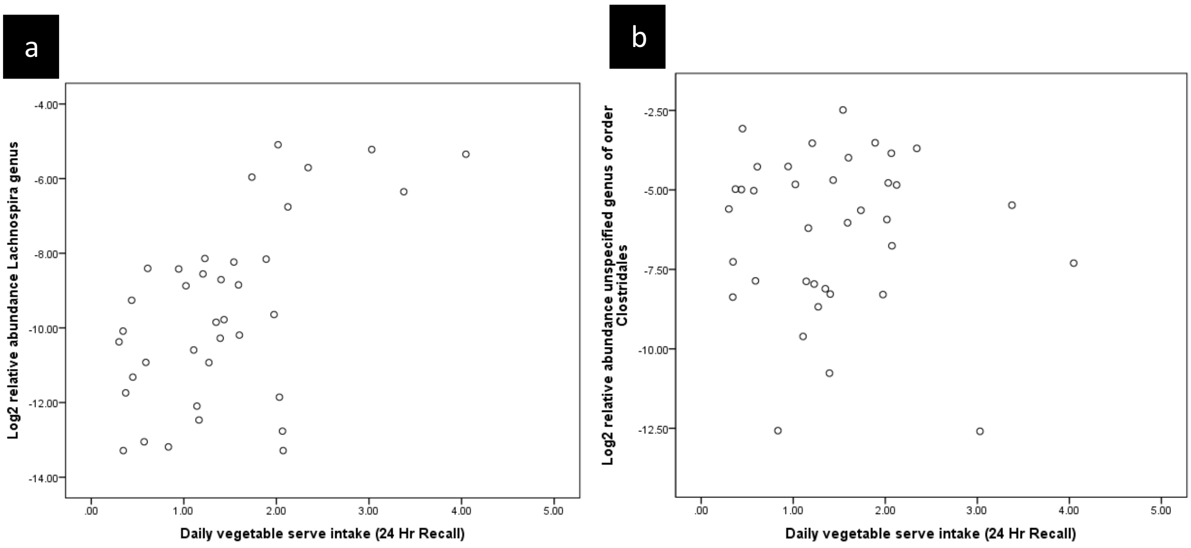

Supplementary Figure 5: Association between fruit intake and relative abundance (log2 (relative abundance + 0.001)) at genus and species level.

a

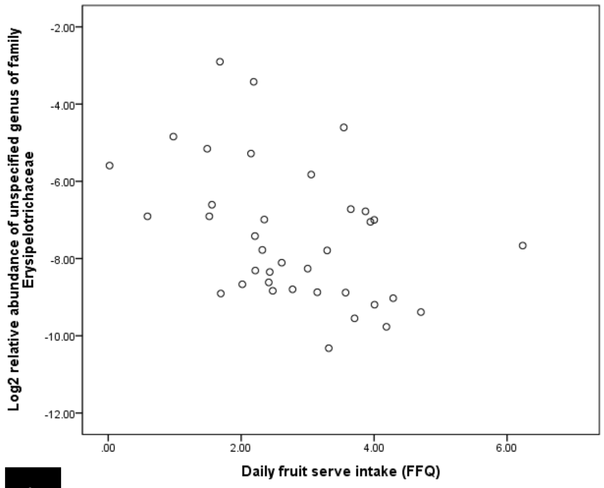

b

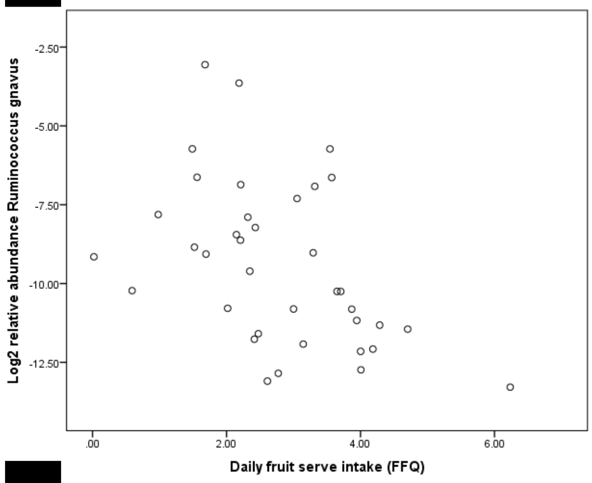

c

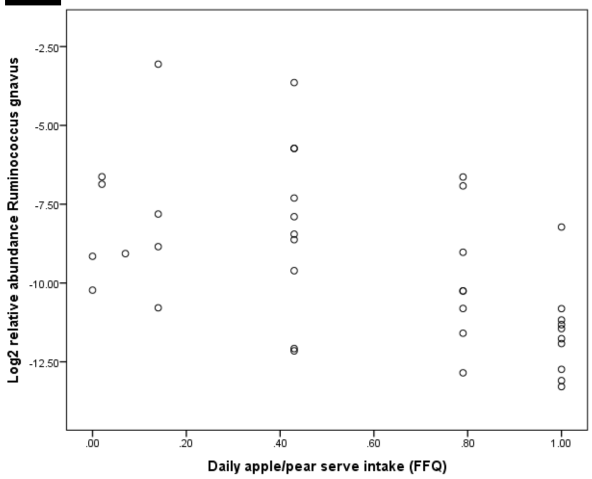

Supplement: Supplementary Information [file srep32385-s1.pdf]
